# Determining the Optimal Cut-Off Values of Serum E2 and FSH for Evaluating the Menopausal Status of Breast Cancer Patients in a Southern Chinese Population

**DOI:** 10.1155/2022/8716160

**Published:** 2022-08-21

**Authors:** Lu Yang, Rucai Li, Mengsi Yu, Fen Huang, Jiangzheng Zeng, Yanda Lu, Changcheng Yang

**Affiliations:** ^1^Department of Medical Oncology, The First Affiliated Hospital of Hainan Medical University, Haikou, Hainan 570102, China; ^2^Department of Radiotherapy, The First Affiliated Hospital of Hainan Medical University, Haikou, Hainan 570102, China; ^3^Department of Clinical Laboratory, The First Affiliated Hospital of Xinjiang Medical University, Urumqi, Xinjiang 830054, China

## Abstract

**Background:**

Chemotherapy-induced amenorrhea (CIA) is one of universal phenomena in breast cancer (BC) patients, and it causes difficulties in evaluating the actual menopausal status which is important for the oncologists to choose appropriate treatment. Currently, serum estradiol (E2) and follicle-stimulating hormone (FSH) levels are the most commonly used clinical parameters for the assessment of menopausal status in BC patients. However, the optimal cut-off points of serum E2 and FSH have little been explored in southern Chinese population.

**Objective:**

This study is aimed to determine the optimal cut-off values of the serum E2 and FSH levels for evaluating the menopausal status of BC patients in a southern Chinese population.

**Methods:**

A retrospective analysis was done among a total of 206 patients with BC from a southern Chinese area. The data of serum E2, FSH, and luteinizing hormone (LH) levels were collected and analyzed for the comparison purpose. The receiver-operating curve (ROC) was generated to assess the specificity and sensitivity of the three biomarkers in discriminating the menopausal status of BC patients. The optimal cut-off values were determined according to the Youden index and then compared with the recommended reference values by the Chinese Anti-cancer Association (CACA) and those recommended by the manufacturers.

**Results:**

The areas under the ROC curves (AUCs) of E2, FSH, and LH were 0.846 (95% CI: 0.790-0.903), 0.781 (95% CI: 0.714-0.847) and 0.608 (95% CI: 0.526-0.690), respectively. The optimal cut-off values were 130.0 pg/mL for E2, 23.325 IU/L for FSH, and 11.625 IU/L for LH with a maximum of the Youden index. When E2, FSH, and LH were used in combination for ROC analysis, the AUC increased to 0.847 (95% CI: 0.790-0.904), which was higher than that of any other biomarker alone. In this study, the sensitivity and specificity of E2 and FSH were 91.6% and 73.70% and 94.4% and 58.6%, respectively, in comparison with 85.0% and 75.80% and 76.6% and 65.7% according to the CACA-recommended cut-off points, or 92.5% and 68.7% and 96.3% and 53.5% according to the manufacturer recommended cut-off points.

**Conclusion:**

Considering the sensitivity and specificity of serum E2 and FSH for assessing the menopausal status, the optimal cut-off values determined in the present study were similar to the manufacturer's recommendations, but obviously superior to the cut-off points suggested by CACA. These cut-off points calculated in this study seem to be valuable in southern Chinese population and might be used by clinicians to make a correct medical decision for BC patients who would benefit from endocrine therapy of aromatase inhibitor (AI).

## 1. Introduction

Breast cancer (BC) is the most common cancer type in Chinese women and is the fifth leading cause of cancer-related death [1, 2]. Nowadays, despite the improvement of diagnostic methods, most BC patients are diagnosed at an advanced stage. Many BC patients should receive adjuvant chemotherapy treatment to reduce the risk of recurrence and metastasis after surgery. Moreover, endocrine therapy is another important adjuvant treatment for BC patients with hormone receptor-positive expression. Currently, tamoxifen (TAM) and aromatase inhibitors (AI) are the most commonly used endocrine therapy drugs for hormone-sensitive BC patients. Notably, AI has been reported to be superior to TAM [3, 4]. But AI drugs could only be used in postmenopausal patients with BC, while TAM has no similar restriction and can be used widely without consideration of the menstrual state. A nationwide multicenter study showed that 62.9% of the BC patients in China were premenopausal at the time of diagnosis, and most of them (81.4%) received chemotherapy as a treatment option [5]. The popularly used chemotherapy drugs in BC are taxanes and anthracycline, both of which tend to induce amenorrhea by interfering with follicular maturation. Evidences have verified that the rate of chemotherapy-induced amenorrhea (CIA) ranged from 35% to 97% in BC patients aged over 40 years [6]. However, amenorrhea is not a reliable biomarker for the determination of menopause, because some BC patients with CIA could recover the normal menstruation after the cessation of chemotherapy. Thus, a large number of premenopausal BC patients have unclear menstruation status after occurrence of CIA, which renders it difficult for oncologists to select endocrine drugs.

Currently, the serum levels of E2 and FSH are being widely used to evaluate the menopausal status of BC patients, which is recommended by several guidelines, including the NCCN and the Chinese Anti-cancer Association (CACA) [7]. Serum E2 and FSH detection have been proved useful for clinical workers to estimate the menopausal status, but these guidelines except CACA offer no cut-off values for them, leading to trouble in using the biomarkers. Though the cut-off points of E2 and FSH have been suggested by the CACA, the points have been found only useful in a limited range. Therefore, it is urgent to define the optimal cut-off values for serum E2 and FSH more widely, especially in southern Chinese areas where this issue has never been paid attention to before, and establish the optimal cut-off points more suitable in the local population.

Here, we retrospectively investigated the serum E2 and FSH levels in a southern Chinese population of 206 patients and generated ROC curves to establish novel cut-off values according to the Youden index, which might help oncologists to estimate whether the BC patients truly enter menopause status and select the best endocrine therapy agents.

## 2. Methods and Materials

### 2.1. Patients and Serum Samples

All breast cancer patients enrolled in this study were diagnosed based on histopathological evaluation at The First Affiliated Hospital of Hainan Medical University. All participants provided verbal or written informed consent for their medical records to be reviewed by our team. Clinical parameters (including patient age, tumor stage and size, nodal stage, estrogen receptor, progesterone receptor, Her-2/neu status, and ethnic) were collected from electronic medical records. Patients with any other malignancy outside the breast were excluded in this study. A total of 206 patients were enrolled in this study, of which 99 patients were pre-menopause and 107 patients were post-menopause. According to a previous study, premenopausal patients were defined as women with regular menses or amenorrhea less than 8 weeks before receiving chemotherapy [8]. Postmenopausal patients were defined as women who aged over 60 years old or underwent bilateral oophorectomy [9, 10]. For eliminating the unfavorable effect of CIA on our data analysis, the pre-menopause group only enrolled the patients with regular menses. All the information collected from BC patients was before any treatment. The detailed clinical characteristics of patients are shown in Table 1. This study was approved by the Ethics Committee of Hainan Medical University in accordance with the Helsinki declaration.

### 2.2. Detection of the Serum Levels of E2, FSH, and LH

2 mL fasting venous blood was collected from all the enrolled BC patients; the blood was centrifuged at a speed of 12000rmp for 5 minutes and then stood for 10 minutes. The upper serum was collected and then tested in time. All serum samples were measured at the Department of Clinical Laboratory, The First Affiliated Hospital, Hainan Medical University. Automatic chemiluminescence immune-analyzer (DXI800, Beckmann, Germany) was applied to detect the serum level of E2, FSH, and LH. Before the test, the instrument should be maintained and checked by professional inspectors on routine maintenance to assure the quality control result is not out of control. Serum bilirubin, hemoglobin, and triglycerides can influence the level of serum hormone, so detection of hormone should avoid lipid and hemolysis [11].

### 2.3. Statistical Analysis

SPSS16.0 software was used for all the statistical analyses. Statistical significance in this study was set at 0.05, and all reported *p* values were 2 sides. Data were presented as means ± standard deviation or median and quartile or range. Nonparametric received operating characteristic (ROC) curves were generated to assess the diagnostic efficiency of serum E2, FSH, and LH in discriminating postmenopausal patients from premenopausal patients. The nonparametric Mann-Whitney *U* test was used to determine the significance of two independent groups.

## 3. Results

### 3.1. Comparison of Serum E2, FSH, and LH Levels in Patients with BC

**Figure 1:**
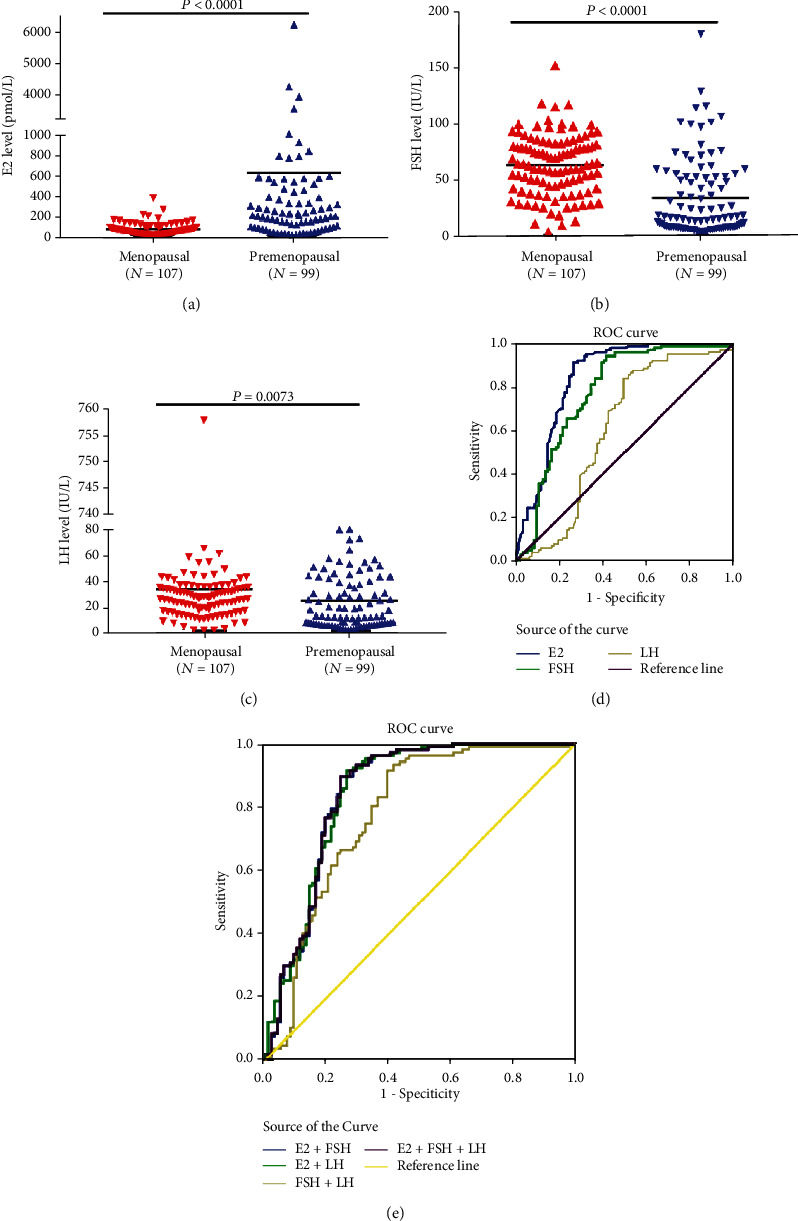
(a) The level of serum E2 was remarkably higher in premenopausal group (*N* =99) than that of menopausal group (*N* =107). (b–c) The serum FSH and LH levels were significantly lower in premenopausal group (*N* =99) compared with menopausal group (*N* =107). (d) ROC curves showed serum E2, FSH, and LH could discriminate premenopausal group from menopausal group and the AUCs were 0.846 (95% CI: 0.790-0.903), 0.781 (95% CI: 0.714-0.847), and 0.608 (95% CI: 0.526-0.690) (*p* < 0.0001, *p* < 0.0001, and *p* = 0.007). (e) When E2, FSH, and LH were used in combination, the AUC of ROC increased to 0.847 (95% CI: 0.790-0.904) (*p* < 0.0001), slightly higher than that of E2 alone.

The levels of serum E2, FSH, and LH were examined in 206 BC patients. The result showed that the median serum E2 concentration was significantly higher in premenopausal group than that of menopausal group (*p* < 0.0001) (Figure 1(a)). For premenopausal patients, the median serum E2 concentration was 242 pmol/L and interquartile range (IQR, P25 and P75) was 111-755 pmol/L; for menopausal patients, the median was 56 pmol/L and IQR was 27-88 pmol/L. Moreover, the serum FSH level was remarkably lower in premenopausal group compared to menopausal group (*p* < 0.0001) (Figure 1(b)). In premenopausal group, the median of serum FSH was 15.52 IU/L (IQR 6.57-50.98 IU/L); for menopausal patients, the median was 61.22 IU/L (IQR 40.79-80.54 IU/L). In addition, the serum LH level in menopausal group was 25.16 IU/L (IQR 15.04-33.21 IU/L), higher than that of the premenopausal group (median 11.45, IQR 5.21-37.81 IU/L) (Figure 1(c)). Collectively, these data suggested that menopausal group had a lower E2 level but higher FSH and LH levels.

### 3.2. Diagnostic Values of Serum E2, FSH, and LH in Discriminating the Menopausal Status of BC Patients

Serum E2, FSH, and LH were demonstrated to be significantly related to menopausal status in BC patients. The role of serum E2, FSH, and LH in differentiating the premenopausal patients from menopausal patients with BC was further evaluated by nonparametric ROC analysis. The AUC for serum E2 was 0.846 (95% CI: 0.790-0.903) (Figure 1(d)). As shown in Table 2, using the cut-off concentration of 130 pmol/L (according to the Youden index), serum E2 produced a sensitivity of 91.6%, a specificity of 73.7%, a positive predictive value (PPV) of 79%, and a negative predictive value (NPV) of 89%. ROC curves were also generated to assess the utility of FSH and LH in differentiating premenopausal group versus menopausal group, and the results showed that the AUCs for FSH and LH were 0.781 (95% CI: 0.714-0.847) and 0.608 (95% CI: 0.526-0.690), respectively (Figure 1(d)). The optimal cut-off points for serum FSH (23.325 IU/L) and LH (11.625 IU/L) were also determined with the maximum of Yuden index. When 23.325 IU/L was used, serum FSH generated a sensitivity of 94.4%, a specificity of 58.6%, a PPV of 71.1%, and a NPV of 90.6%. However, serum LH had a relatively lower specificity (84.1%) and specificity (50.5%). In addition, E2, FSH, and LH were used in combination and the AUC was 0.847, slightly higher than that of E2 alone (Figure 1(e) and Table 2). Taken together, these data strongly suggested that the serum E2 and FSH levels could be used for discriminating premenopausal patients from menopausal patients in our study.

### 3.3. Comparison of the Diagnostic Efficiencies of Serum E2, FSH, and LH according to Distinct Cut-Off Points

For the comparison purpose, the specificity, sensitivity, PPV, NPV, and accuracy of serum E2, FSH, and LH were calculated according to different cut-off points for discrimination of premenopausal status from menopausal status. As shown in Table 3, when used the cut-off points determined by the present study, the sensitivity, specificity, and accuracy of serum E2 determination were 91.6%, 73.7%, and 83.0%, respectively. When used the cut-off points recommended by the CACA or manufacturer, the sensitivity, specificity, and accuracy of serum E2 were 85.0%, 75.8%, and 80.6% and 92.5%, 68.7%, and 81.1%, respectively. The cut-off values defined by the current study produced a relatively higher diagnostic accuracy. Similarly, the sensitivity and specificity of serum FSH obtained by our data were 94.4% and 58.6%, respectively, comparable with those obtained by the CACA or manufacturer recommended cut-off points (76.6% and 65.7% and 96.3% and 53.3%, respectively) with a higher accuracy (77.2% vs 71.4%, 75.7%). For serum LH, there is no cut-off value recommended by CACA, while the cut-off values defined by us or offered by the manufacturer produced the similar diagnostic performance. In general, the diagnostic performance of cut-off pointed defined in this study was comparable with those offered by the manufacturers, but seemed to be superior to those recommended by the CACA.

## 4. Discussion

Currently, endocrine therapy is extensively used in the treatment of BC patients. Endocrine therapy is mainly applied for adjuvant treatment and the management of recurrence in BC patients with hormone sensitivity [12, 13]. The use of endocrine therapeutic drugs targeting estrogen receptor (ER) has observably decreased the recurrence and improved survival time in BC patients with different stages. At present, tamoxifen and aromatase inhibitors (AIs) are commonly applied endocrine therapy agents. AIs are more and more chosen to treat the postmenopausal BC patients with hormone receptor-positive, which was demonstrated superior to tamoxifen [14–17]. However, AIs could not be used in women with premenopausal status unless in combination with ovarian function suppression [18, 19]. The menopausal status of CIA patients is unclear and difficult to assess, which makes it difficult for oncologists to choose correct endocrine drugs. Therefore, it is needed to estimate these patients whether truly enter menopause. Menopause is defined as the permanent cessation of menses with permanent decrease in ovarian estrogen synthesis [20–22]. But amenorrhea is not a reliable biomarker for assessing the menopausal status of BC patients who underwent chemotherapy and CIA is not equal to menopause [8]. In current practice, several guidelines have recommended that the menstruation status of patients with CIA could be evaluated by detecting serum levels of FSH and E2, which have been popularly used worldwide. However, due to various reasons, such as different detection methods and different population, most important is that most of the guidelines have no optimal cut-off values for serum E2 and FSH. In 2012, the CACA offered the expert consensus for judging menopause status and clinical application of AIs in Chinese premenopausal BC patients after adjuvant treatment. In this consensus, the cut-off values of FSH>40 IU/L and E2<110 pmol/L (or<30 pg/mL) were suggested with a notice of adjustment should be made after further clinical data accumulation [23]. Nevertheless, few studies have been reported since the census formulation, and few data have been accumulated so far; thus, the cut-off values have not been adjusted. It has never been explored whether those two cut-off values are suitable for the southern Chinese population and whether novel cut-off values needed to be determined for adjustment of the CACA recommendation. Here, we performed a retrospective study to establish optimal clinical cut-off values for serum E2 and FSH as diagnostic markers in discrimination of pre-menopause from postmenopausal status in BC patients, in an attempt to help the oncologists make correct decision to select endocrine therapy drugs for BC patients with unclear menstruation status.

In the present study, our results showed that serum E2 concentrations were observably increased in BC patients with premenopausal status and could discriminate premenopausal status from postmenopausal status. Meanwhile, the serum FSH levels were found to be significantly higher in postmenopausal group compared with premenopausal group. Importantly, the level of serum FSH exerted an effective role in differentiating premenopausal status from postmenopausal status. This is consistent with the previous studies which have demonstrated that the serum concentrations of E2 and FSH closely were correlated with the menstruation status of BC patients [24, 25]. By ROC analysis, we found that the AUCs for serum E2 and FSH were 0.846 and 0.781, respectively, both greater than 0.7. The AUC increased to 0.847 when E2 and FSH were analyzed in combination. When used to differentiate BC patients with premenopausal status from postmenopausal ones, serum E2 and FSH had a sensitivity of 91.6% and 94.4% and a specificity of 73.7% and 58.6%, respectively. These excellent performance characteristics have also been reported in other studies, for example, high AUC of 0.939 for serum E2 and 0.971 for serum FSH, which was reported by Meng et al. [26]. Thus, our data further confirmed the utility of serum E2 and FSH measurement in distinguishing pre-menopause from postmenopausal status in BC patients.

In further analysis, the optimal cut-off points of serum E2 and FSH were determined by using ROC curves according to the Youden index with the maximum. In our study, the optimal E2 cut-off point was 130 pmol/L, different from the 110 pmol/L (CACA recommended) and the 146.8 pmol/L (manufacturer's recommendation). Importantly, in our analysis, the diagnostic accuracy of serum E2 was higher when used the cut-off point of 130 pmol/L compared with the recommended cut-off point of 110 pmol/L or 146.8 pmol/L. Similarly, the optimal cut-off point of serum FSH in our data was 23.325 IU/L, different from those recommended by the CACA (40 IU/L) or manufacturer (16.74 IU/L). In the comparison analysis of diagnostic values, most of the variables of serum FSH using the cut-off value of 23.32 IU/L were superior to those obtained from the cut-off values recommended by the CACA. The reasons for these differences might exist in the fact that different study populations and different measurement methods, especially the CACA expert consensus, only offering the cut-off values without mentioning the detection method and kits. It is well known that different methods and kits for one detection index will generate distinct reference ranges [27, 28]. Thus, it is not surprising that the cut-off values determined by our study were obviously different with those from the CACA expert consensus. Therefore, the optimal cut-off values of serum E2 and FSH should be determined individually to be more suitable for local population. Our present study provided the first data concerning the optimal cut-off values of serum E2 and FSH in a southern Chinese population and our determined cut-off values were more useful in comparison with the CACA recommendations. But it should be noticed that our results are obtained retrospectively in a single center and further large-scale multiple-center prospective studies are required to verify this result. In addition, all the pre-menopause patients should be recorded on the first day of menstrual bleeding, as well as the day of blood sample collection, and determined the menstrual cycle phase of sampling time. It will help to establish more accuracy in identifying optimal cut-off values of E2 and FSH serum levels.

In conclusion, our data suggested that the optimal cut-off points for serum E2 and FSH were 130 pmol/L and 23.325 IU/L, respectively, in the present study population, a southern Chinese population. Using these individual cut-off values, the serum E2 and FSH had better diagnostic performance in discriminating BC patients under premenopausal status from postmenopausal status, thus helping clinical oncologists select optimal endocrine therapy drugs.

## Figures and Tables

**Table 1:** Clinical parameters of breast cancer patients.

Clinical parameters	Premenopausal group (*n* =99)	Menopausal group (*n* =107)
Age(median/range)	44 (30-56)	63 (45-80)
*Histological type*		
Invasive duct cancer	35	45
Others	64	62
*Lymph node metastasis*		
(+)	23	31
(-)	56	58
Unknown	20	18
*ER status*		
(+)	76	76
(-)	23	31
*PR status*		
(+)	65	66
(-)	34	41
*Her-2 status*		
(+)	39	28
(-)	60	79
*Ki 67*		
<20%	28	35
≥20%	71	72
E2 level(median/range)	242 (10-6105) pmol/L	56 (5-364) pmol/L
FSH level(median/range)	15.52 (2-177) IU/L	61.22 (2.4-149.5) IU/L
LH level(median/range)	11.45 (0.62-102.7) IU/L	25.16 (0.32-157) IU/L
*Ethnic group*		
Han ethnicity	95	107
Others	4	0

**Table 2:** The diagnostic values of serum E2, FSH, and LH in evaluating menopausal status of breast cancer patients.

Biomarkers	AUC	Sensitivity	Specificity	PPV	NPV	Accuracy
E2	0.846	91.6%	73.7%	79.0%	89.0%	83.0%
FSH	0.781	94.4%	58.6%	71.1%	90.6%	77.2%
LH	0.608	84.1%	50.5%	64.7%	74.6%	67.9%
E2 + FSH	0.846	89.7%	75.8%	79.3%	87.2%	85.0%
E2 + LH	0.847	91.6%	73.7%	80.3%	89.0%	82.6%
FSH + LH	0.781	91.6%	60.6%	71.5%	89.9%	76.7%
E2 + FSH + LH	0.847	90.7%	75.8%	80.2%	87.2%	83.5%

PPV: positive predictive value; NPV: negative predictive value.

**Table 3:** Comparison of the diagnostic performance of E2, FSH, and LH according to different cut-off points.

Biomarker	Cut-off points	Sensitivity	Specificity	PPV	NPV	Accuracy
E2	130 pmol/L^∗^	91.6%	73.7%	79.0%	89.0%	83.0%
	146.8 pmol/L^†^	92.5%	68.7%	76.2%	89.5%	81.1%
	110 pmol/L^‡^	85.0%	75.8%	79.1%	82.4%	80.6%
FSH	23.325 IU/L^∗^	94.4%	58.6%	71.1%	90.6%	77.2%
	16.74 IU/L^†^	96.3%	53.5%	69.1%	93.0%	75.7%
	40 IU/L^‡^	76.6%	65.7%	70.7%	72.2%	71.4%
LH	11.625 IU/L^∗^	84.1%	50.5%	64.7%	74.6%	67.9%
	10.87 IU/L^†^	86.0%	47.5%	63.9%	75.8%	67.5%
	No recommend					

^∗^Cut-off points calculated by ROC analysis in the present study. ^†^Manufacture recommended cut-off points. ^‡^CACA-recommended cut-off points.

## Data Availability

Data available on request.

## Abbreviations

CIA: Chemotherapy-induced amenorrhea
E2: Estradiol
FSH: Follicle-stimulating hormone
TAM: Tamoxifen
AI: Aromatase inhibitor
BC: Breast cancer
ROC: Receiver-operating characteristics curve
CACA: Chinese anti-cancer association
AUC: Area under the curve
LH: Luteinizing hormone.
